# A novel *SLC20A2* nonsense variant and mechanistic studies of primary brain calcification

**DOI:** 10.1371/journal.pone.0346635

**Published:** 2026-04-17

**Authors:** Yi Li, Lamei Yuan, Han Chen, Wen Zheng, Hongbo Xu, Zhijian Yang, Dan He, Hao Deng

**Affiliations:** 1 Department of Laboratory Medicine, the Third Xiangya Hospital, Central South University, Changsha, Hunan, China; 2 Research Center of Medical Experimental Technology, the Third Xiangya Hospital, Central South University, Changsha, Hunan, China; 3 Center for Experimental Medicine, the Third Xiangya Hospital, Central South University, Changsha, Hunan, China; 4 Department of Neurology, the Third Xiangya Hospital, Central South University, Changsha, Hunan, China; 5 Department of Pathology, Hunan Aerospace Hospital, Changsha, Hunan, China; 6 Disease Genome Research Center, Central South University, Changsha, Hunan, China; Zhengzhou University, CHINA

## Abstract

Primary brain calcification (PBC) is a rare neurodegenerative disease featured by bilateral brain calcifications and exhibiting high phenotypic and genetic heterogeneity. The clinical manifestations mainly include movement disorders, cognitive deficits, and neuropsychiatric symptoms. In this study, a novel heterozygous nonsense variant, c.1669C > T [p.(Gln557*)], in the solute carrier family 20 member 2 gene (*SLC20A2*), encoding type III sodium-dependent inorganic phosphate transporter 2 (PiT2), was identified in a Han-Chinese family with PBC using whole exome sequencing and Sanger sequencing. Bioinformatics analysis predicted the variant’s deleterious effect. The cellular function impacts of the p.Gln557* variant and three other common PBC-related *SLC20A2* variants, p.Ser113*, p.Ala585Thr, and p.Ser601Trp, were subsequently revealed. Subcellular localization analysis showed that the PiT2-Q557*, A585T, and S601W mutants mainly distributed in the plasma membrane and cytosol, whereas the PiT2-S113* mutant showed a diffuse distribution throughout the cells. All four investigated variants significantly impaired cellular inorganic phosphate transport activity. The protein mislocalization-inducing p.Ser113* variant likely causes haploinsufficiency, while the p.Gln557*, p.Ala585Thr, and p.Ser601Trp variants may lead to full or partial loss of function, or exert a dominant-negative effect. Cells expressing PiT2 mutants all exhibited inhibited proliferative and migratory activities, along with enhanced apoptosis. These *SLC20A2* variants probably impair critical cellular functions, potentially providing an explanation for the neurological symptoms observed in PBC patients. These findings further broaden *SLC20A2* variant spectrum and provide valuable mechanistic insights into the pathogenesis of *SLC20A2*-associated PBC.

## Introduction

Primary brain calcification (PBC) is a rare and incurable monogenic neurodegenerative disorder characterized by bilateral symmetrical calcifications major in the basal nuclei [1]. Historically, this condition has been referred to as Fahr’s disease, idiopathic basal ganglia calcification (IBGC), and primary familial brain calcification [2,3]. Clinically, PBC is associated with a broad spectrum of motor and neuropsychiatric symptoms, such as dystonia, parkinsonism, ataxia, tremor, muscle spasms, cramps, dizziness, headaches, epilepsy, chorea, frontal-subcortical cognitive dysfunction, dementia, bipolar disorder, and psychosis [4,5]. Its pathological hallmark is vascular and pericapillary calcification in the brain, primarily composed of calcium phosphate deposits [6]. The estimated prevalence of PBC ranges from 2.1 to 6.6 per 1000 individuals [7,8]. PBC can be sporadic or inherited in various patterns, with typical onset age between the fourth and sixth decades of life, although considerable intra- and inter-familial variability exists [9]. The diagnosis is complicated by its reliance on neuroimaging for diagnosis, incomplete penetrance, and substantial underlying genetic heterogeneity [10].

To date, nine causative genes for PBC have been cataloged in Online Mendelian Inheritance in Man (OMIM) database, including four autosomal dominant genes and five autosomal recessive genes [11–14]. The solute carrier family 20 member 2 gene (*SLC20A2*, MIM 158378), encoding type III sodium-dependent inorganic phosphate transporter 2 (PiT2), was identified as the first PBC gene in 2012, and pathogenic variants in this gene were considered as the most common cause of PBC, recorded as IBGC1 in OMIM (MIM 213600). Variable missense, nonsense, splicing, deletion, and duplication/insertion variants have been revealed [6,15].

In this study, a novel heterozygous nonsense variant, c.1669C > T [p.(Gln557*)], in the *SLC20A2* gene (NM_001257180.2), was identified by whole exome sequencing (WES) and Sanger sequencing in a Han-Chinese patient presenting with clinical characteristics of PBC. To further investigate the potential impacts of PBC-related *SLC20A2* variants, we performed cell-based experiments of the identified novel nonsense variant c.1669C > T (p.Gln557*) and three additional common reported variants: the functionally characterized pathogenic missense variant c.1802C > G (p.Ser601Trp) was used as a positive control [6,16], while the representative nonsense variant c.338C > G (p.Ser113*) and missense variant c.1753G > A (p.Ala585Thr) were selected for distinct locations in PiT2 functional domains [8,10]. The different subcellular localization of the mutants and their impaired cellular inorganic phosphate (Pi) uptake supported the involvement of haploinsufficiency (or loss-of-function) or dominant-negative effect in disease pathogenesis. Furthermore, these variants were found to inhibit cell proliferation and migration while promoting apoptosis.

## Materials and methods

### Subjects and clinical evaluations

A 49-year-old Han-Chinese male with PBC was recruited for the present study from Changsha, China, as well as a 60-year-old unrelated healthy male without relative disease and family history as a control. The patient diagnosed with PBC was evaluated by two neurologists based on medical history and a series of examinations, such as head computed tomography (CT), and neurological and serologic examinations, which included routine blood tests, liver and renal functions, electrolytes, parathyroid hormone (PTH), and myocardial enzymes. After obtaining all the participants’ written informed consent, peripheral venous blood samples were collected. The study was conducted from December 26, 2018, to December 31, 2024, in accordance with the principles of the Declaration of Helsinki and with the approval from the Institutional Review Board of the Third Xiangya Hospital of Central South University (Changsha, China).

### Whole exome sequencing

The genomic DNA (gDNA) of the proband (II:1) was separated from the blood sample by the phenol-chloroform method, and WES was subsequently performed for screening genetic variants (BGI-Shenzhen, Shenzhen, China). Qualified gDNA was randomly fragmented via the Covaris technique. After end repairing, A-tailing reactions, and adapter ligation, the captured fragments were then amplified by ligation-mediated polymerase chain reaction, followed by purification and enrichment. Captured fragments were amplified by cyclization, and then DNA nanoballs were generated by rolling circle amplification, which were further sequenced on the DNBSEQ platform [17].

### Variant analysis

The pipeline for WES data is summarized as follows. High-quality clean reads were generated by SOAPnuke (v2.1.0) and were then aligned to the human reference genome (GRCh37/hg19) using the Burrows-Wheeler Aligner software (v0.7.17). Duplicate reads were marked and eliminated by the MarkDuplicates tool of Genome Analysis Toolkit (GATK, v4.1.4.1), and the sequencing base quality values were recalibrated by the GATK BaseRecalibrator and ApplyBQSR for high quality. The potential variants, including single nucleotide polymorphisms (SNPs) and insertions/deletions (InDels), were revealed and filtered through the GATK HaplotypeCaller, SelectVariants, and VariantFiltration tools, which were further annotated with Annodb software [18]. Databases including the Single Nucleotide Polymorphism database Build 156, the 1000 Genomes Project, the National Heart, Lung, Blood Institute-Exome Sequencing Project 6500, the Exome Aggregation Consortium, ClinVar, and the in-house BGI exome database (BGI-Shenzhen, Shenzhen, China) with 2,471 Chinese controls were integrated [19]. Potential candidate variants were further filtered by databases including the Genome Aggregation Database (http://gnomad.broadinstitute.org/), the China Metabolic Analytics Project (http://www.mbiobank.com/info/), Human Gene Mutation Database (https://www.hgmd.cf.ac.uk/ac/index.php), and the Leiden Open Variation Database (https://www.lovd.nl/), as well as our in-house exome database with 965 controls [20].

### Sanger sequencing

The filtered candidate PBC-associated variant was further verified by the polymerase chain reaction amplification and Sanger sequencing. The site-specific primers used were designed by Primer3 (https://primer3.ut.ee/), 5’-TACAAACAAGGCGGGGTAAC-3’ and 5’-TGTCTCCTCCTCCCTGTGAC-3’, with the specificity verified *in silico* using Primer–BLAST (https://www.ncbi.nlm.nih.gov/tools/primer-blast/).

### Bioinformatics analysis

Online prediction software programs, which include Functional Analysis through Hidden Markov Models (FATHMM, http://fathmm.biocompute.org.uk/), MutPred-LOF (http://mutpred2.mutdb.org/mutpredlof/), MutationTaster2021 (https://www.genecascade.org/MutationTaster2021/), Combined Annotation Dependent Depletion (CADD, http://cadd.gs.washington.edu/score), and Genomic Evolutionary Rate Profiling (GERP), were applied to assess the variant’s effect [21]. The Basic Local Alignment Search Tool (BLAST, http://blast.ncbi.nlm.nih.gov/Blast.cgi) was used to perform multiple protein sequence alignment. The three-dimensional structure models of wild-type (WT) and the variant-type protein sequences were predicted by SWISS-MODEL (http://swissmodel.expasy.org) and visualized using Visual Molecular Dynamics software (v1.9.3). The identified variant was further interpreted using American College of Medical Genetics and Genomics and Association for Molecular Pathology (ACMG/AMP) guidelines.

### Cell culture, plasmid construction, and transfection

The human neuroblastoma SH-SY5Y cell line was employed to model neuronal functions due to the catecholaminergic neuronal property, human origin, and ease of maintenance [22,23]. The human embryonic kidney 293 T (HEK293T) cell was employed as a heterologous expression system for functional characterization of proteins, given the high transfection efficiency and widespread use in evaluating PBC-related genes [12,16]. SH-SY5Y (Procell, China) and HEK293T (Procell, China) cells were cultured in Dulbecco’s modified Eagle’s medium (Gibco, USA) supplemented with 10% fetal bovine serum (Gibco, USA) and incubated in an incubator at 37℃ in a humidified 5% CO_2_ atmosphere. The *SLC20A2* WT and variant-type (c.338C > G, c.1669C > T, c.1753G > A, and c.1802C > G) cDNA were synthesized (YouBio, China) and transferred into the pCDH-CMV-Flag-EF1-Puro plasmid using restriction endonucleases (Thermo Scientific, USA), EcoRⅠ and BamHI. All plasmid constructs were verified by Sanger sequencing. Transfections were performed using Lipofectamine 2000 reagent (Invitrogen, USA) when cells reached approximately 80% confluence, following the manufacturer’s instructions.

### Immunofluorescence

Upon reaching 50% confluence, the cultured SH-SY5Y and HEK293T cells expressing WT or variant-type PiT2 in a specific dish were fixed with 4% paraformaldehyde for 20 min at room temperature (RT), followed by three washes with phosphate-buffered saline (PBS). Cells were then permeabilized with 0.1% Triton X-100 (BioFroxx, Germany) for 10 min and blocked with 5% bovine serum albumin (BSA) in PBS for 1 h at RT, followed by incubation with the Flag Tag Mouse Monoclonal Antibody (1:100, Beyotime, China) at 4℃ overnight. After three washes with PBS, the cells were incubated for 1 h with the secondary antibody, CY3 Conjugated AffiniPure Goat Anti-Mouse IgG (H + L) (1:300, Boster, China). Finally, the nuclei were stained with 4’,6-diamidino-2-phenylindole (DAPI, Biosharp, China) for 5 min. Images were acquired with a Zeiss LSM 800 confocal laser scanning microscope (Carl Zeiss, Germany).

### Western blotting

The transfected cells were lysed using the cell lysis buffer supplemented with the proteinase inhibitor phenylmethylsulfonyl fluoride (Solarbio, China) on ice for 10 min, and then, they were scraped, sonicated, and centrifuged at 15,500 × g for 15 min at 4℃. The extracted proteins were separated by 12% sodium dodecyl sulfate-polyacrylamide gel and blotted onto 0.22 μm polyvinylidene difluoride membrane (Millipore, USA). After blocked with 5% BSA for 1 h at RT, the membrane was incubated with the mouse Flag antibody (1:2000, Beyotime, China) or rabbit β-actin antibody (1:5000, Proteintech, China) at 4℃ overnight. After three washes, the membrane was further incubated with the secondary antibody, horseradish peroxidase (HRP)-conjugated goat anti-mouse or anti-rabbit IgG (H + L) (1:5000, Proteintech, China) for 1 h at RT. Finally, the membrane was incubated with an enhanced chemiluminescence HRP substrate kit (Biosharp, China) and the image was obtained with a ChemiDoc MP Imaging System (Bio-Rad, USA).

### Pi uptake evaluation

The measurement of Pi concentration in the cell culture medium was applied to indirectly reflect cellular Pi uptake, in which a higher residual Pi concentration indicates lower uptake [24,25]. This alternative method avoids the regulatory complexities and safety hazards of radioactive ^32^Pi uptake assay. SH-SY5Y or HEK293T cells expressing WT or variant-type PiT2 were seeded in a 96-well plate at an initial density of 1 × 10^4^ cells per well. After 48 h, the culture medium was collected, and the Pi concentration was quantified using a Malachite Green Phosphate Detection Kit (Beyotime, China) according to the manufacturer’s instructions. Absorbance of the generated complex reflecting the Pi concentration, which is inversely proportional to Pi transport activity and uptake, was measured at 630 nm by a microplate spectrophotometer (Bio-Tek, USA). Three independent assays were performed in 96-well plates.

### Cell viability assay

To examine the effect of *SLC20A2* variants on cell proliferation, cell viability was evaluated with Cell Counting Kit-8 (CCK-8, Biosharp, China) according to manufacturer’s instructions. SH-SY5Y or HEK293T cells were placed in 96-well plates at 5 × 10^3^ or 2 × 10^3^ cells per well, and the CCK-8 reagent was added into each well and incubated for 2.5 h or 1 h at 37℃. The absorbance of each well at the indicated timepoints was measured at 450 nm by a microplate spectrophotometer (Bio-Tek, USA).

### Wound scratch assay

Cell migration ability was evaluated by a wound scratch assay. When the cells in 6-well plates reached 90% confluence, a sterile 200 μL pipette tip was used to gently scrape uniform wound scratches in each well. After two washes with PBS, the cells were cultured in the serum-deprived medium. The wound images were captured (10 × magnification) at 0 h and 48 h using an inverted microscope (Carl Zeiss, Germany), and scratch areas were measured using ImageJ software (National Institutes of Health, USA). The relative migration rate was expressed as the percentage of wound closure, calculated as: [(Area_0 h_ - Area_48 h_) / Area_0 h_] × 100% [26,27].

### Cell apoptosis assay

To assess the effects of *SLC20A2* variants on the cell apoptosis profile, an apoptosis assay was performed. Upon reaching 90% confluence, the cells in 6-well plates were collected and washed twice with cold PBS. In the dark, the cells were resuspended with Binding Buffer, and further stained with Annexin V-FITC and propidium iodide (PI) for 10 min using the Annexin V-FITC/PI Cell Apoptosis Detection Kit (Yeasen, China) following the manufacturer’s instructions. The prepared samples were then evaluated by a BD LSRFortessa^™^ flow cytometry (BD Biosciences, USA), and the data were analyzed with FlowJo software (FlowJo, USA).

### Statistical analysis

In each cellular assay, all data were presented as the mean ± standard error of the mean from three or four representative experiments. In each independent experiment, three technical replicates were applied to ensure the reliability and reproducibility, and the obtained mean of replicates was used. Statistical graphs and statistical analyses were completed by GraphPad Prism v.9.0 software (GraphPad, USA). Differences in independent groups were analyzed by one-way or two-way Analysis of Variance (ANOVA) and Tukey’s multiple comparison test. Before applying ANOVA, data were tested for normality and homogeneity of variance by the Shapiro-Wilk and Brown-Forsythe test, respectively. The convention was as follows: * *P* < 0.05, ** *P* < 0.01, *** *P* < 0.001, and **** *P* < 0.0001.

## Results

### Clinical findings of the PBC patient

**Fig 1:**
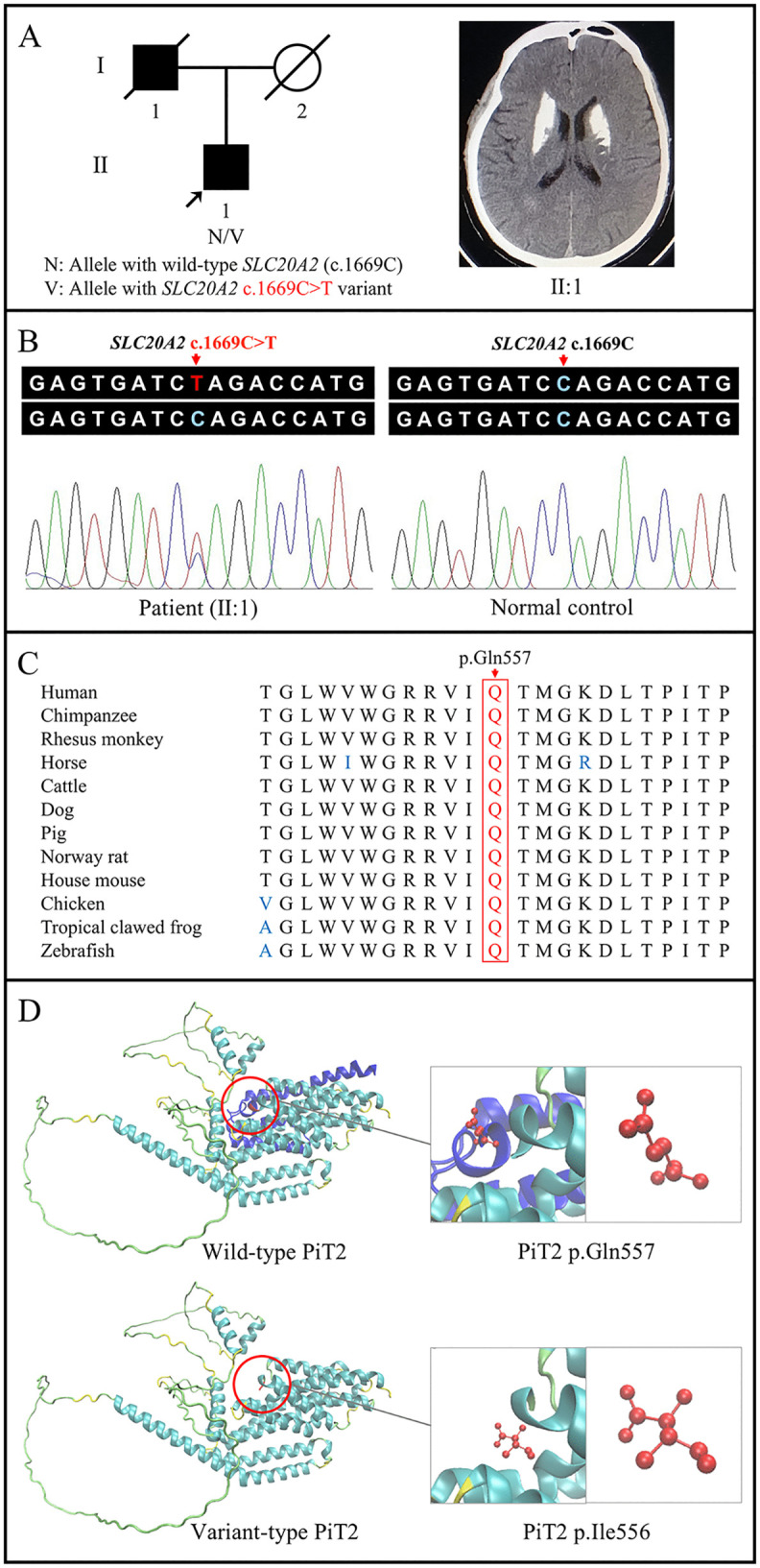
Pedigree, brain calcifications, and variant analysis of a Han-Chinese family with PBC. **(A)** Pedigree of the PBC family and bilateral basal nuclei calcifications on the head computed tomography scan of the patient (II:1). A square represents a male, and a circle represents a female. An empty symbol represents an unaffected member, a filled symbol represents an affected member, and the symbol with a slash represents a deceased individual. The proband is indicated by an arrow. **(B)** The *SLC20A2* sequence with the heterozygous c.1669C > T [p.(Gln557*)] variant in the patient (II:1) and the normal *SLC20A2* sequence in the normal control revealed by Sanger sequencing. **(C)** Conservation analysis of PiT2 protein with the residue at the mutated position (corresponding to human p.Gln557) indicated by a red rectangle. **(D)** Cartoon models of wild-type PiT2 (dark blue: residues at position 558 to 652) and variant-type PiT2, with the glutamine at position 557 of wild-type PiT2 (p.Gln557) and the last residue of variant-type PiT2, isoleucine at position 556 (p.Ile556), colored in red, further displayed in ball-and-stick models. PBC, primary brain calcification; PiT2, type III sodium-dependent inorganic phosphate transporter 2; *SLC20A2*, the solute carrier family 20 member 2 gene.

The proband (Ⅱ:1) complained of headache, vomit, and vertigo. His medical history indicated chronic gastritis. Speech disturbance, dystonia, or gait disturbances were not found in the proband. No remarkable changes were observed in laboratory analysis, with normal serum levels of calcium, phosphate, and PTH, excluding calcium dysregulation and other metabolic disorders. Head CT scan showed bilateral symmetrical calcification in the basal nuclei (Fig 1A), with no evidence of other brain abnormalities.

### Identification of a novel *SLC20A2* variant

A total of 88.14 million raw reads were generated from WES of the proband. In the obtained 86.19 million clean reads, approximately 99.99% were aligned to the human reference genome. The average sequencing depth of the target region was 118.93 × , with 98.34% and 84.54% covered at least 20× and 50 × , respectively. A total of 127,572 SNPs and 24,256 InDels were detected in the proband. Following a comprehensive filtering process, a novel heterozygous *SLC20A2* nonsense variant, c.1669C > T [p.(Gln557*)], was considered as the sole candidate variant of PBC, absent in public variant and disease databases, as well as in-house exome databases (ACMG/AMP evidence: PM2, Table 1). No other potential variants were shown in known PBC genes. The heterozygous nonsense variant was further confirmed in the proband by Sanger sequencing (ACMG/AMP: PVS1), but absent in the normal control (Fig 1B).

**Table 1:** Analysis of the *SLC20A2* gene variant identified in our patient with PBC.

Category		Information
Nucleotide change		c.1669C > T
Amino acid change		p.(Gln557*)
Transcript reference sequence		NM_001257180.2
Exon		9
Zygosity		Heterozygote
Variant type		Nonsense
Single Nucleotide Polymorphism database Build 156		No
Allele frequency	1000 Genomes Project	No
	National Heart, Lung, and Blood Institute-Exome Sequencing Project 6500	No
	Exome Aggregation Consortium	No
	Genome Aggregation Database	No
	China Metabolic Analytics Project	No
	In-house exome database	No
ClinVar		No
Human Gene Mutation Database		No
Leiden Open Variation Database		No
Functional Analysis through Hidden Markov Models (score)		Damaging (0.98506)
MutPred-LOF (score)		Pathogenic (0.62984)
MutationTaster2021		Deleterious
Combined Annotation Dependent Depletion (phred score)		Deleterious (42)
Genomic Evolutionary Rate Profiling (score)		Conserved (5.71)
American College of Medical Genetics and Genomics and Association for Molecular Pathology variant interpretation guidelines		Pathogenic (PVS1 + PM2 + PP3)

PBC, primary brain calcification; PM, pathogenic moderate; PP, pathogenic supporting; PVS, pathogenic very strong; *SLC20A2*, the solute carrier family 20 member 2 gene.

### Bioinformatics analysis of *SLC20A2* variant

Bioinformatics tools, including FATHMM, MutPred-LOF, MutationTaster2021, CADD, and GERP, predicted the *SLC20A2* gene variant, c.1669C > T [p.(Gln557*)], to be damaging (ACMG/AMP: PP3, Table 1). Sequence alignment showed that the affected residue at position 557 (p.Gln557) was highly conserved across multiple species (Fig 1C). The three-dimensional protein structure model revealed the substantial conformational alteration caused by the variant (Fig 1D). According to the ACMG/AMP criteria, the identified nonsense variant was classified as “pathogenic” (PVS1 + PM2 + PP3, Table 1).

### Effects of *SLC20A2* variants on PiT2 expression

**Fig 2:**
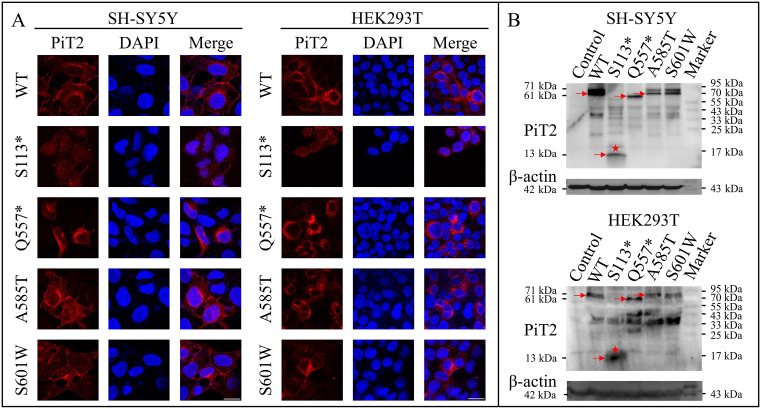
The expression of WT and variant-type PiT2 (S113*, Q557*, A585T, and S601W) in SH-SY5Y and HEK293T cells. **(A)** Immunofluorescence of WT and variant-type PiT2 in SH-SY5Y and HEK293T cells. Cells were stained with anti-Flag/CY3 (red) antibodies and DAPI (blue). Scale bar = 20 μm. **(B)** Western blotting of WT and variant-type PiT2 in SH-SY5Y and HEK293T cells. The arrows indicate the corresponding PiT2 proteins: WT and two variant-type PiT2 (PiT2-WT, A585T, and S601W, ~ 71 kDa), PiT2-S113* (~13 kDa), PiT2-Q557* (~61 kDa), and β-actin (42 kDa), with the molecular mass indicated on the left of image. The red star indicates PiT2-S113*, with a doubled total protein load. Control, cells transfected with pCDH-CMV-Flag-EF1-Puro; DAPI, 4’,6-diamidino-2-phenylindole; PiT2, type III sodium-dependent inorganic phosphate transporter 2; WT, wild-type, cells transfected with pCDH-CMV-SLC20A2-Flag-EF1-Puro.

The *SLC20A2* WT and variant-type (c.338C > G, c.1669C > T, c.1753G > A, and c.1802C > G) plasmid constructs, all conjugated with a Flag tag expressing at the PiT2 C-terminus (the DYKDDDDK sequence), were successfully transfected into SH-SY5Y and HEK293T cells. To evaluate whether the variants affect the subcellular localization, immunofluorescence staining on cultured cells was applied. Compared with PiT2-WT localized to the plasma membrane and cytosol, PiT2-Q557*, A585T, and S601W mutants showed a similar distribution, whereas PiT2-S113* exhibited a diffuse distribution in the cytosol and nucleus (Fig 2A). Similarly, compared with PiT2-WT, the PiT2-Q557*, A585T, and S601W mutants showed no significant differences in nuclear-to-cytoplasmic ratio of fluorescence intensity (all *P* > 0.05), while the PiT2-S113* mutant exhibited a significantly increased ratio (*P* < 0.001). The expression of Flag-tagged WT and variant-type PiT2 was verified by Western blotting. Notably, the p.S113* mutant exhibited a lower level, requiring a doubled total protein load to achieve detectable signals (Fig 2B).

### Effects of *SLC20A2* variants on Pi transport activity of PiT2

**Fig 3:**
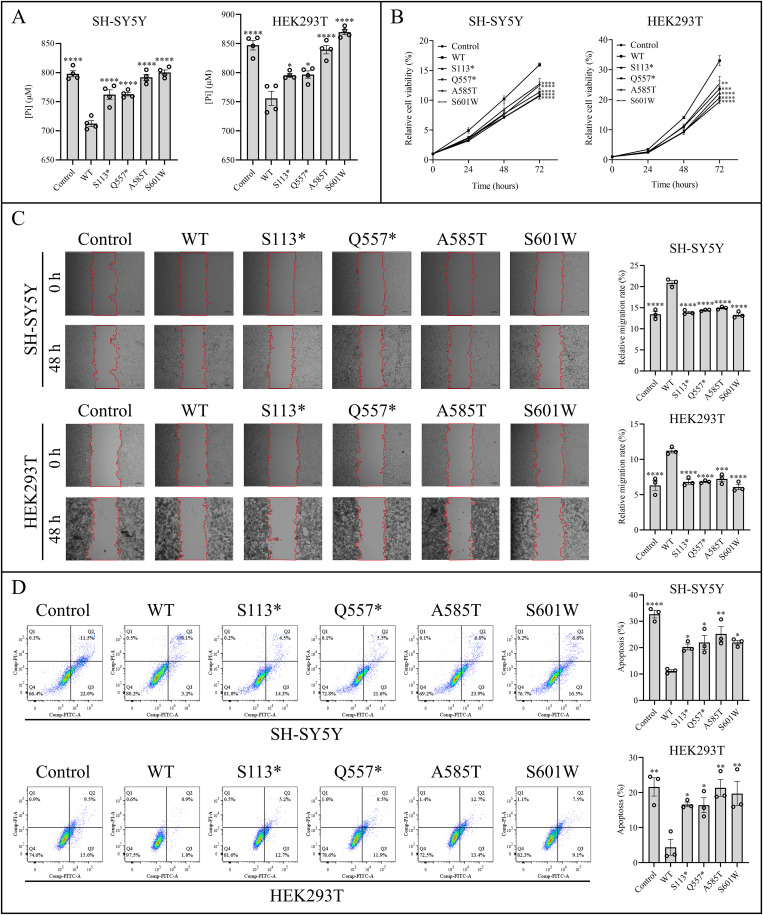
The Pi transport activities and cellular effects of WT and variant-type PiT2 (S113*, Q557*, A585T, and S601W) in SH-SY5Y and HEK293T cells. **(A)** Pi uptake assay by detecting [Pi] in culture medium after 48 h of cell culture. Compared to the control group, the WT group showed decreased Pi concentration, and the four groups expressing variant-type PiT2 exhibited higher Pi concentration than the WT group, indicating a decreased Pi uptake. Statistics are shown as the mean ± SEM for four representative experiments. * *P* < 0.05 and **** *P* < 0.0001 (one-way ANOVA with Tukey’s multiple comparison test). **(B)** Cell viability determined by CCK-8 assay. Compared to the control group, the WT group showed increased cell viability, and the four groups expressing variant-type PiT2 presented diminished viability than the WT group. Statistics are shown as the mean ± SEM for three representative experiments. ** *P* < 0.01, *** *P* < 0.001, and **** *P* < 0.0001 (two-way ANOVA with Tukey’s multiple comparison test). **(C)** Cell migration measured by the wound scratch assay. Representative images of the scratch at 0 h and 48 h. Compared to the control group, the WT group exhibited enhanced migratory capacity, and the four groups expressing variant-type PiT2 showed a marked inhibition of migration than the WT group. Scale bar = 200 μm. Statistics are shown as the mean ± SEM for three representative experiments. *** *P* < 0.001 and **** *P* < 0.0001 (one-way ANOVA with Tukey’s multiple comparison test). **(D)** The cell apoptosis evaluated by flow cytometry. Compared to the control group, the WT group showed decreased apoptosis, and the four groups expressing variant-type PiT2 exhibited increased apoptosis than the WT group. Statistics are shown as the mean ± SEM for three representative experiments. * *P* < 0.05, ** *P* < 0.01, and **** *P* < 0.0001 (one-way ANOVA with Tukey’s multiple comparison test). ANOVA, Analysis of Variance; CCK-8, Cell Counting Kit-8; Control, cells transfected with pCDH-CMV-Flag-EF1-Puro; Pi, inorganic phosphate; [Pi], Pi concentration; PiT2, type III sodium-dependent inorganic phosphate transporter 2; SEM, standard error of the mean; *SLC20A2*, the solute carrier family 20 member 2 gene; WT, wild-type, cells transfected with pCDH-CMV-SLC20A2-Flag-EF1-Puro.

To determine the potential impacts of *SLC20A2* variants on Pi transport in SH-SY5Y and HEK293T cells, the Pi uptake assay was applied. The medium Pi concentration of groups expressing variant-type PiT2 was significantly higher than the WT group, demonstrating impaired Pi transport activities in mutant groups, and the expression of PiT2-WT promoted the Pi uptake compared to the control group (all *P* < 0.05, Fig 3A).

### Cellular effects of *SLC20A2* variants

To further explore the potential cellular effects of *SLC20A2* variants on SH-SY5Y and HEK293T cells, the cell viability assay, wound scratch assay, and cell apoptosis assay were applied. Groups expressing variant-type PiT2 presented diminished cell viability than the WT group, and compared to the control group, the expression of PiT2-WT increased cell viability (all *P* < 0.05, Fig 3B). The wound scratch assay results demonstrated that groups expressing variant-type PiT2 showed a marked inhibition of cell migration compared to the WT group, and the expression of PiT2-WT enhanced migratory capacity compared to the control group (all *P* < 0.05, Fig 3C). The cell apoptosis assay revealed that groups expressing variant-type PiT2 exhibited significantly increased apoptosis relative to the WT group, and the expression of PiT2-WT decreased apoptosis compared to the control group (all *P* < 0.05, Fig 3D).

## Discussion

Patients with PBC typically present with brain calcification and normal serum calcium and phosphate levels, but the Pi level in the cerebrospinal fluid (CSF) may be significantly elevated, especially in patients with *SLC20A2* variants [28]. There may be a close association between *SLC20A2* variants and disrupted brain Pi homeostasis. Such imbalance is regarded as a key pathogenic mechanism of PBC [29]. In addition, Pi is necessary for many important functions, including energy metabolism, signal transduction, nucleic acid synthesis, cell survival, muscle contractility, neurological functions, and mineralizing process [30]. Pi dysregulation therefore induces the profound pathological consequences.

**Fig 4:**
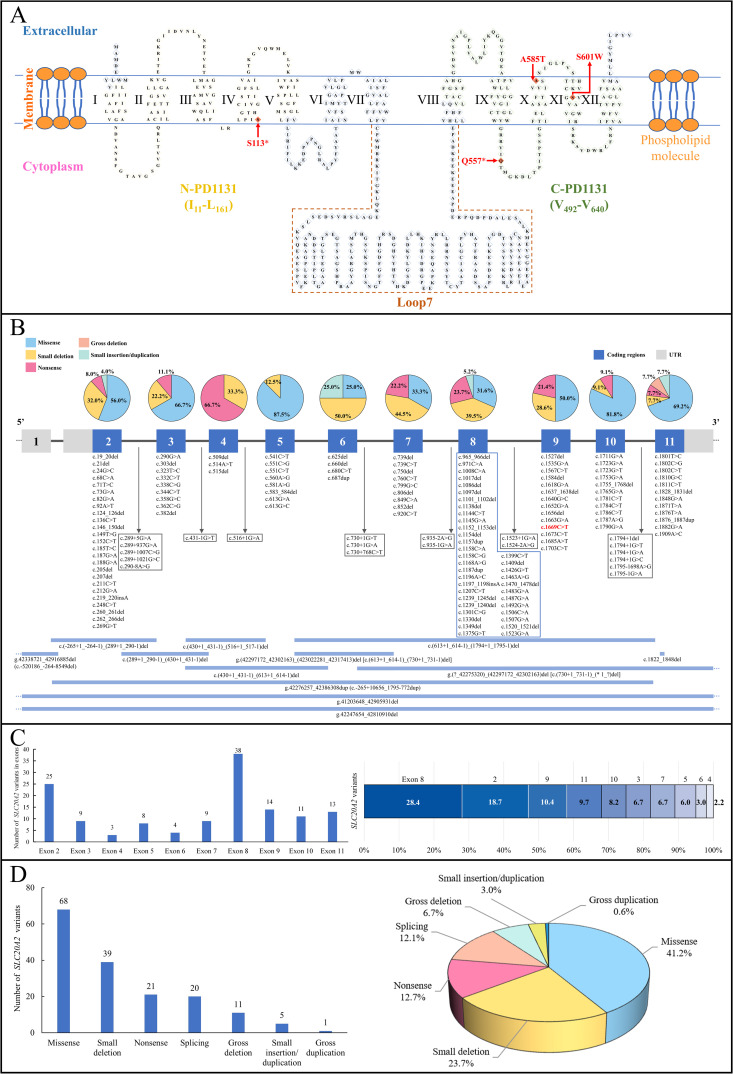
The topology of protein PiT2 and the *SLC20A2* gene variants associated with PBC reported in previous research and this study. **(A)** The topology of protein PiT2 with the studied variants marked and the transmembrane regions (I-XII) indicated (UniProtKB: Q08357). **(B)** Schematic distribution of *SLC20A2* variants associated with PBC. The numbered boxes represent exons in *SLC20A2.* The pie chart above the exons described the proportion of different variant types. The bottom long strips depict the gross deletion or duplication variants involving *SLC20A2*. The variant in red (c.1669C > T) is the novel variant identified in this study. **(C)** The number and proportion of *SLC20A2* variants associated with PBC in exons. **(D)** The number and proportion of *SLC20A2* variants associated with PBC in different variant types, in which gross deletion or duplication refers to large deletion or duplication over 20 bp. PBC, primary brain calcification; PiT2, type III sodium-dependent inorganic phosphate transporter 2; *SLC20A2*, the solute carrier family 20 member 2 gene; UTR, untranslated regions.

The Pi transporter PiT2, encoded by the *SLC20A2* gene on chromosome 8p11.21, is a 652-amino acid, 12-pass transmembrane protein. It is widely expressed in multiple tissues, with high expression in neurons, astrocytes, and vascular endothelial cells of the brain [31,32]. The protein features two ProDom motifs, designated N-PD1131 (I_11_-L_161_) and C-PD1131 (V_492_-V_640_), and an intracellular loop region (loop7) (Fig 4A). Both elements, ProDom related to Pi-binding ability and loop7 determining the phosphorylation-mediated membrane localization, play crucial roles in Pi transport activity [33].

As the first PBC gene, the *SLC20A2* gene is the most common pathogenic gene, with heterozygous variants accounting for over 60% of genetically verified cases [15]. To date, at least 165 *SLC20A2* variants linked to PBC have been identified, without prominent hotspots (Fig 4B–4D, S1 Table). Variants vary from point mutations to gross rearrangements, including missense substitutions (41.2%), small deletions (23.7%), nonsense variants (12.7%), splicing variants (12.1%), gross deletions (6.7%), small insertions/duplications (3.0%), and gross duplications (0.6%).

In this study, a novel heterozygous *SLC20A2* nonsense variant, c.1669C > T [p.(Gln557*)], was identified in a Han-Chinese patient with bilateral symmetrical calcification of the basal nuclei in the absence of metabolic abnormalities. The patient’s main clinical manifestation was chronic headache, a recognized nonmotor symptom of PBC, and no other related neurological abnormalities were complained. The CSF Pi level was unknown as the patient declined lumbar puncture due to its invasive nature. The nonsense p.(Gln557*) variant, affecting the conserved residue positioned in C-PD1131 domain of PiT2, led to the loss of a large part of functionally critical domain. Integrated evidence, including the absence in all the screened databases and consistent predictions from multiple bioinformatics tools, further supported the pathogenic role of this variant.

As previously reported, missense mutants involving the critical loop7 region, such as PiT2-T390A and PiT2-S434W, exhibited decreased membrane localization [34]. Notably, in this study, the nonsense PiT2-S113* mutant, truncating within N-PD1131 and lacking the entire loop7 and C-PD1131, showed a diffuse intracellular distribution and an increased nuclear-to-cytoplasmic fluorescence ratio, consistent with loss of plasma membrane localization. This mislocalization precluded its interaction with PiT2-WT. In conjunction with its reduced expression, these findings support a probable haploinsufficiency mechanism through nonsense-mediated decay, similar to that proposed for the previously reported PiT2-R467* mutant, which lacks a portion of loop7 and the entire C-PD1131 [35].

Haploinsufficiency (or loss-of-function) and dominant-negative effects are implicated in the molecular pathogenesis of *SLC20A2*-PBC [36,37]. The N-terminal nonsense variant c.338C > G (p.Ser113*) likely acts through haploinsufficiency. The C-terminal nonsense variant c.1669C > T (p.Gln557*) and two missense variants, c.1753G > A (p.Ala585Thr) and c.1802C > G (p.Ser601Trp), may lead to full or partial loss of function, or exert a dominant-negative effect. The severe defect, resulting in low residual transport capacity, correlates with the more serious clinical phenotypes observed in a subset of *SLC20A2*-patients, including earlier disease onset and more pronounced symptoms. A few patients with c.1753G > A (p.Ala585Thr) and c.1802C > G (p.Ser601Trp) were reported to be asymptomatic, which may be modified by genetic background, environmental influence, epigenetic factors, etc. (Table 2) [6,8,10,35,38–43]. Based on our data and the information in the literature, we speculate that the mechanisms of the *SLC20A2* pathogenic variants in PBC may be categorized as follows. Variants causing protein mislocalization usually lead to haploinsufficiency. Variants that preserve normal localization and induce milder phenotypes or no symptoms generally result in declined or full loss of function, while those leading to preserved normal localization but more severe phenotypes may exert dominant-negative effects. Owing to the significant genetic and clinical heterogeneity, a precise genotype-phenotype correlation for *SLC20A2*-associated PBC remains to be elucidated.

**Table 2:** The summary of relevant PBC cases with *SLC20A2* variants analyzed in this study.

Variant No.	Variant	Variant type	Case No.	Ethnicity	AAO/AAE (yrs)	Gender	Clinical symptoms	Head CT scan	References
1	c.338C > G, p.(Ser113*)	Nonsense	1	NA	NA/68	F	Dystonia and asymmetric parkinsonism	Bilateral symmetric basal nuclei calcification	[10]
			2	Caucasian	53/65	M	Focal unilateral chorea	Basal nuclei, thalamus, and dentate nuclei calciﬁcation	[38,39]
			3	NA	NA	F	NA	Brain calciﬁcation^#^	[39]
2	c.1669C > T, p.(Gln557*)	Nonsense	1	Chinese	NA/49	M	Headache, vomit, and vertigo	Basal nuclei calciﬁcation	Our study^†^
3	c.1753G > A, p.(Ala585Thr)	Missense	1	NA	NA	NA	NA	NA	[40]
			2	NA	37/38	M	Headache and right arm tremor	Bilateral basal nuclei calcification	[41]
			3	African	NA	F	Dementia and parkinsonism	Basal nuclei, thalamus, dentate nuclei, and cerebral cortex calciﬁcation	[39]
			4	Chinese	26/30	F	Vertigo	6 (TCS)	[42]
			5	Chinese	NA	F	Asymptomatic	38 (TCS)	[8]
4	c.1802C > G, p.(Ser601Trp)	Missense	1	Chinese	73/75	F	Parkinsonism and cerebral infarction	Basal nuclei, inferior thalamus, and cerebral cortex calciﬁcation	[6]
			2	Chinese	NA/65	M	Asymptomatic	Basal nuclei, inferior thalamus, and cerebral cortex calciﬁcation	
			3	Chinese	NA/62	F	Asymptomatic	Basal nuclei calciﬁcation	
			4	Chinese	NA/43	M	Asymptomatic	Basal nuclei and inferior thalamus calciﬁcation	
			5	Chinese	NA/40	F	Asymptomatic	Basal nuclei and inferior thalamus calciﬁcation	
			6	Chinese	NA/36	M	Asymptomatic	Basal nuclei and inferior thalamus calciﬁcation	
			7	Chinese	1/22	F	Repetitive epilepsy, mental retardation, and developmental delay	Basal nuclei, inferior thalamus, cerebral cortex, and cerebellar hemisphere calciﬁcation	
			8	Chinese	0.5/20	F	Repetitive epilepsy, severe mental, retardation, dysarthria, ataxia, and developmental delay	Basal nuclei, inferior thalamus, cerebral cortex, and cerebellar hemisphere calciﬁcation	

^#^Localization of brain calciﬁcation was not available.

^†^The variant, c.1669C > T [p.(Gln557*)], was the novel variant identified in our study.

AAE, age at examination; AAO, age at onset; CT, computed tomography; F, female; M, male; NA, not available; PBC, primary brain calcification; *SLC20A2*, the solute carrier family 20 member 2 gene; TCS, total calcification score; yrs, years.

PBC patients with the studied *SLC20A2* variants mainly presented neurological symptoms, including headache, epilepsy, parkinsonism, and mental retardation (Table 2) [13]. The protein PiT2 was highly expressed in the brain, especially in neurons, and our cellular functional data demonstrated that the variants significantly disrupted the Pi homeostasis in both neuronal (SH-SY5Y) and non-neuronal (HEK293T) models. Compared with the control group, expression of PiT2-WT significantly enhanced cellular proliferative and migratory capacities, and markedly inhibited apoptosis. All variants substantially impaired these processes. The findings were complementarily supported by discoveries of significantly increased neuronal apoptosis in *Slc20a2* knockdown mice and in neuronal models derived from *SLC20A2*-PBC patients [44,45]. Collectively, these findings suggested that pathogenic *SLC20A2* variants may impair cellular functions, potentially providing an explanation for the neurological symptoms observed in PBC patients. Additionally, the obtained results of Pi uptake assay may be confounded by factors such as cell number, metabolic state, phosphate secretion, and cell death, particularly given the unexpected cellular effects induced by the variants themselves. Therefore, the assay can only indirectly reflect the cellular Pi uptake function. Accordingly, in-depth investigations with more precise approaches, including protein-normalized radiolabeled ^32^Pi uptake assays, as well as studies in brain endothelial cells, neurons, and *in vivo* systems, are warranted to clarify the mechanistic link between these cellular phenotypes and the neurological manifestations and to further explore the underlying pathogenic mechanisms of *SLC20A2*-associated PBC [44,46,47].

The *Slc20a2* knockout mice displayed a similar calcification phenotype and elevated CSF Pi levels as *SLC20A2*-PBC patients, supporting the crucial role of PiT2 in regulating brain Pi homeostasis [48,49]. Currently, PBC treatment primarily remains symptomatic. Recently, intracerebroventricular injection of antisense oligonucleotides enhanced *SLC20A2* expression and inhibited brain calcification in human *SLC20A2* variant knockin mice, offering a new disease-modifying treatment strategy [50,51]. Constructing more genetic deficiency animal models of PBC for experimental therapy will contribute to accelerating the development of precision therapies.

## Conclusions

In conclusion, a novel heterozygous *SLC20A2* nonsense variant, c.1669C > T [p.(Gln557*)], was identified in a Han-Chinese family with PBC. Functional analyses revealed that this nonsense variant impaired cellular Pi transport activity and critical cellular processes. These effects were consistent with those induced by the c.338C > G (p.Ser113*), c.1753G > A (p.Ala585Thr), and c.1802C > G (p.Ser601Trp) variants, while the c.338C > G (p.Ser113*) variant additionally showed subcellular mislocalization of PiT2. The observed alterations and discrepancies suggest distinct pathogenic mechanisms underlying *SLC20A2*-PBC: protein mislocalization-inducing p.Ser113* variant likely causes disease through haploinsufficiency, while the p.Gln557*, p.Ala585Thr, and p.Ser601Trp variants may lead to full or partial loss of function, or exert a dominant-negative effect. This study expands the *SLC20A2* gene variant spectrum and provides deeper mechanistic insights into PBC pathogenesis, offering valuable implications for developing novel therapeutic strategies.

## Supporting information

S1 Table The reported variants involving in the *SLC20A2* gene related to PBC. (PDF)

S1 Raw Images Raw Western blotting images for Fig 2B. (PDF)
