# Supplementary material for: A novel SLC20A2 nonsense variant and mechanistic studies of primary brain calcification
Source: PLoS One. 2026 Apr 17;21(4):e0346635. doi: 10.1371/journal.pone.0346635 (PMC13089887; doi:10.1371/journal.pone.0346635)
Supplement: S1 Table — (PDF) [file pone.0346635.s001.pdf]

Supplementary Table 1 The reported variants involving the *SLC20A2* gene related to PBC.

No.	Sequence change#	Variant type§	Involved regions	References
1	g.42338721_42916885del (c.-520186_-264-8549del)/p.?	Gross deletion	Partial *SLC20A2* (exon 1-intron 1) and other 6 genes	[1]
2	c.(-265+1_-264-1)_(289+1_290-1)del/p.?	Gross deletion	Partial *SLC20A2* (intron 1-intron 2)	[2]
3	g.42276257_42386308dup (c.-265+10656_1795-772dup)/p.?	Gross duplication	Partial *SLC20A2* (intron 1-intron 10)	[3]
4	g.42247654_42810910del/p.?	Gross deletion	Whole *SLC20A2* (exon 1-exon 11) and other 7 genes	[4]
5	g.41203648_42905931del/p.?	Gross deletion	Whole *SLC20A2* (exon 1-exon 11) and other 21 genes	[5]
6	c.19_20del/p.Leu7Valfs*48	Small deletion/ frameshift	*SLC20A2* exon 2	[6]
7	c.21del/p.(Leu7Phefs*10)	Small deletion/ frameshift	*SLC20A2* exon 2	[7]
8	c.24G>C/p.(Trp8Cys)	Missense	*SLC20A2* exon 2	[8]
9	c.68C>A/p.Ser23Tyr	Missense	*SLC20A2* exon 2	[6]
10	c.71T>C/p.(Val24Ala)	Missense	*SLC20A2* exon 2	[9]
11	c.73G>A/p.Gly25Ser	Missense	*SLC20A2* exon 2	[6]
12	c.82G>A/p.(Asp28Asn)	Missense	*SLC20A2* exon 2	[10]
13	c.92A>T/p.(Asn31Ile)	Missense	*SLC20A2* exon 2	[11]
14	c.124_126del/p.(Val42del)	Small deletion	*SLC20A2* exon 2	[12]
15	c.136C>T/p.(Gln46*)	Nonsense	*SLC20A2* exon 2	[13]
16	c.146_150del/p.(Ile49Serfs*5)	Small deletion/ frameshift	*SLC20A2* exon 2	[9]
17	c.149T>G/p.(Leu50*)	Nonsense	*SLC20A2* exon 2	[14]
18	c.152C>T/p.(Ala51Val)	Missense	*SLC20A2* exon 2	[15]
19	c.185T>C/p.(Leu62Pro)	Missense	*SLC20A2* exon 2	[10]
20	c.187G>A/p.(Gly63Ser)	Missense	*SLC20A2* exon 2	[16]
21	c.188G>A/p.(Gly63Asp)	Missense	*SLC20A2* exon 2	[16]
22	c.205del/p.Thr69Profs*9	Small deletion/ frameshift	*SLC20A2* exon 2	[6]
23	c.207del/p.(Ile70Phefs*8)	Small deletion/ frameshift	*SLC20A2* exon 2	[9]
24	c.211C>T/p.(Arg71Cys)	Missense	*SLC20A2* exon 2	[17]
25	c.212G>A/p.(Arg71His)	Missense	*SLC20A2* exon 2	[15]
26	c.219_220insA/p.(Ile74Asnfs*3)	Small insertion/ frameshift	*SLC20A2* exon 2	[18]
27	c.248C>T/p.Thr83Met	Missense	*SLC20A2* exon 2	[6, 19]

No.	Sequence change[#]	Variant type[§]	Involved regions	References
28	c.260_261del/p.(Leu87Hisfs*6)	Small deletion/ frameshift	*SLC20A2* exon 2	[15]
29	c.262_266del/p.(Met88Trpfs*4)	Small deletion/ frameshift	*SLC20A2* exon 2	[20]
30	c.269G>T/p.(Gly90Val)	Missense	*SLC20A2* exon 2	[21]
31	c.(289+1_290-1)_(430+1_431-1)del/p.Gly97_Ile143del	Gross deletion	Partial *SLC20A2* (intron 2-intron 3)	[6]
32	c.289+5G>A/p.?	Splicing	*SLC20A2* intron 2	[14]
33	c.289+937G>A/p.Ser98Argfs*9	Splicing/ frameshift	*SLC20A2* intron 2	[6]
34	c.289+1007C>G/p.?	Splicing	*SLC20A2* intron 2	[22]
35	c.289+1021G>C/p.?	Splicing	*SLC20A2* intron 2	[22]
36	c.290-8A>G/p.Gly97Alafs*163	Splicing/ frameshift	*SLC20A2* intron 2	[6, 14]
37	c.290G>A/p.(Gly97Asp)	Missense	*SLC20A2* exon 3	[14]
38	c.303del/p.(Trp101Cysfs*3)	Small deletion/ frameshift	*SLC20A2* exon 3	[23]
39	c.323T>C/p.(Leu108Pro)	Missense	*SLC20A2* exon 3	[24]
40	c.332C>T/p.(Pro111Leu)	Missense	*SLC20A2* exon 3	[9]
41	c.338C>G/p.(Ser113*)	Nonsense	*SLC20A2* exon 3	[4]
42	c.344C>T/p.(Thr115Met)	Missense	*SLC20A2* exon 3	[15]
43	c.358G>C/p.(Gly120Arg)	Missense	*SLC20A2* exon 3	[25]
44	c.362C>G/ p.(Ser121Cys)	Missense	*SLC20A2* exon 3	[12]
45	c.382del/(p.Val128Serfs*43)	Small deletion/ frameshift	*SLC20A2* exon 3	[14]
46	c.(430+1_431-1)_(516+1_517-1)del/p.?	Gross deletion	Partial *SLC20A2* (intron 3-intron 4)	[2]
47	c.(430+1_431-1)_(613+1_614-1)del/p.Val144_Pro204del	Gross deletion	Partial *SLC20A2* (intron 3-intron 5)	[2, 6]
48	c.431-1G>T/p.?	Splicing	*SLC20A2* intron 3	[26]
49	c.509del/p.(Leu170*)	Nonsense	*SLC20A2* exon 4	[27]
50	c.514A>T/p.(Lys172*)	Nonsense	*SLC20A2* exon 4	[27]
51	c.515del/p.Lys172Argfs*20	Small deletion/ frameshift	*SLC20A2* exon 4	[28]
52	c.516+1G>A/p.Val144Glyfs*85	Splicing/ frameshift	*SLC20A2* intron 4	[21]
53	c.541C>T/p.(Arg181Trp)	Missense	*SLC20A2* exon 5	[14, 29]
54	c.551C>G/p.(Pro184Arg)	Missense	*SLC20A2* exon 5	[30]
55	c.551C>T/p.(Pro184Leu)	Missense	*SLC20A2* exon 5	[26]
56	c.560A>G/p.(Tyr187Cys)	Missense	*SLC20A2* exon 5	[31]
57	c.581A>G/p.(Asn194Ser)	Missense	*SLC20A2* exon 5	[26]

No.	Sequence change#	Variant type§	Involved regions	References
58	c.583_584del/p.(Val195Leufs*62)	Small deletion/ frameshift	*SLC20A2* exon 5	[27]
59	c.613G>A/p.(Val205Met)	Missense	*SLC20A2* exon 5	[32]
60	c.613G>C/p.(Val205Leu)	Missense	*SLC20A2* exon 5	[33]
61	g.(42297172_42302163)_(423022281_ 42317413)del [c.(613+1_614-1)_(730+1_731-1)del]/p.(Val205_Thr243del)	Gross deletion	Partial *SLC20A2* (intron 5-intron 6)	[5, 23]
62	c.(613+1_614-1)_(1794+1_1795-1)del/p.?	Gross deletion	Partial *SLC20A2* (intron 5-intron 10)	[34]
63	c.625del/p.(Val209Phefs*7)	Small deletion/ frameshift	*SLC20A2* exon 6	[35]
64	c.660del/p.Phe220Leufs*23	Small deletion/ frameshift	*SLC20A2* exon 6	[6]
65	c.680C>T/p.(Ala227Val)	Missense	*SLC20A2* exon 6	[36]
66	c.687dup/p.(Val230Cysfs*28)	Small duplication/ frameshift	*SLC20A2* exon 6	[29]
67	c.730+1G>T/p.?	Splicing	*SLC20A2* intron 6	[14]
68	c.730+1G>A/p.?	Splicing	*SLC20A2* intron 6	[9]
69	g.(?_42275320)_(42297172_42302163)del [c.(730+1_731-1)_(*1_?)del]/p.?	Gross deletion	Partial *SLC20A2* (intron 6-3' UTR)	[22]
70	c.730+768C>T/p.?	Splicing	*SLC20A2* intron 6	[23]
71	c.739del/p.Gln247Lysfs*71	Small deletion/ frameshift	*SLC20A2* exon 7	[6]
72	c.739C>T/p.(Gln247*)	Nonsense	*SLC20A2* exon 7	[14]
73	c.750del/p.(Ala251Leufs*67)	Small deletion/ frameshift	*SLC20A2* exon 7	[31]
74	c.760C>T/p.Arg254*	Nonsense	*SLC20A2* exon 7	[6, 27]
75	c.799G>C/p.(Glu267Gln)	Missense	*SLC20A2* exon 7	[5]
76	c.806del/p.(Pro269Glnfs*49)	Small deletion/ frameshift	*SLC20A2* exon 7	[37]
77	c.849C>A/p.Ser283Arg	Missense	*SLC20A2* exon 7	[6]
78	c.852del/p.(Ile285Serfs*33)	Small deletion/ frameshift	*SLC20A2* exon 7	[38]
79	c.920C>T/p.(Pro307Leu)	Missense	*SLC20A2* exon 7	[39]
80	c.935-2A>G/p.?	Splicing	*SLC20A2* intron 7	[13]
81	c.935-1G>A/p.Gly312Valfs*8	Splicing/ frameshift	*SLC20A2* intron 7	[10]
82	c.965_966del/p.(Val322Glufs*92)	Small deletion/ frameshift	*SLC20A2* exon 8	[40]
83	c.971C>A/p.(Ser324*)	Nonsense	*SLC20A2* exon 8	[41]
84	c.1008C>A/p.His336Gln	Missense	*SLC20A2* exon 8	[6]

No.	Sequence change[#]	Variant type[§]	Involved regions	References
85	c.1017del/p.(Ser339Argfs*116)	Small deletion/ frameshift	*SLC20A2* exon 8	[42]
86	c.1086del/p.(His362Glnfs*93)	Small deletion/ frameshift	*SLC20A2* exon 8	[43]
87	c.1097del/p.(Gly366Alafs*89)	Small deletion/ frameshift	*SLC20A2* exon 8	[44]
88	c.1101_1102del/p.(Glu368Glyfs*46)	Small deletion/ frameshift	*SLC20A2* exon 8	[45]
89	c.1138del/p.(Leu380Cysfs*75)	Small deletion/ frameshift	*SLC20A2* exon 8	[31]
90	c.1144C>T/p.(Arg382*)	Nonsense	*SLC20A2* exon 8	[46]
91	c.1145G>A/p.(Arg382Gln)	Missense	*SLC20A2* exon 8	[27]
92	c.1152_1153del/p.(Asn384Lysfs*30)	Small deletion/ frameshift	*SLC20A2* exon 8	[42]
93	c.1154del/p.(Ser385Ilefs*70)	Small deletion/ frameshift	*SLC20A2* exon 8	[37]
94	c.1157dup/p.Tyr386*	Nonsense	*SLC20A2* exon 8	[6, 9]
95	c.1158C>A/p.(Tyr386*)	Nonsense	*SLC20A2* exon 8	[42]
96	c.1158C>G/p.Tyr386*	Nonsense	*SLC20A2* exon 8	[47]
97	c.1168A>G/p.Thr390Ala	Missense	*SLC20A2* exon 8	[6]
98	c.1187dup/p.(Pro397Alafs*18)	Small duplication/ frameshift	*SLC20A2* exon 8	[14]
99	c.1196A>C/p.(His399Pro)	Missense	*SLC20A2* exon 8	[16]
100	c.1197_1198insA/p.(Ala400Serfs*15)	Small insertion/ frameshift	*SLC20A2* exon 8	[9]
101	c.1207C>T/p.(Arg403*)	Nonsense	*SLC20A2* exon 8	[14]
102	c.1239_1245del/p.Glu414Trpfs*39	Small deletion/ frameshift	*SLC20A2* exon 8	[6]
103	c.1239_1240del/p.Ser413Argfs*25	Small deletion/ frameshift	*SLC20A2* exon 8	[6]
104	c.1301C>G/p.(Ser434Trp)	Missense	*SLC20A2* exon 8	[45]
105	c.1330del/p.(Glu444Argfs*11)	Small deletion/ frameshift	*SLC20A2* exon 8	[9]
106	c.1349del/p.(Gly450Alafs*5)	Small deletion/ frameshift	*SLC20A2* exon 8	[48]
107	c.1375G>T/p.(Glu459*)	Nonsense	*SLC20A2* exon 8	[49]
108	c.1399C>T/p.Arg467*	Nonsense	*SLC20A2* exon 8	[6, 15]
109	c.1409del/p.(Pro470Leufs*38)	Small deletion/ frameshift	*SLC20A2* exon 8	[12]
110	c.1426G>T/p.(Glu476*)	Nonsense	*SLC20A2* exon 8	[14]
111	c.1463A>G/p.(His488Arg)	Missense	*SLC20A2* exon 8	[14]

No.	Sequence change[#]	Variant type[§]	Involved regions	References
112	c.1470_1478del/p.(Gln491_Leu493del)	Small deletion	*SLC20A2* exon 8	[10]
113	c.1483G>A/p.(Ala495Thr)	Missense	*SLC20A2* exon 8	[50]
114	c.1487G>A/p.(Cys496Tyr)	Missense	*SLC20A2* exon 8	[36]
115	c.1492G>A/p.(Gly498Arg)	Missense	*SLC20A2* exon 8	[12]
116	c.1506C>A/p.(His502Gln)	Missense	*SLC20A2* exon 8	[27]
117	c.1507G>A/p.(Gly503Ser)	Missense	*SLC20A2* exon 8	[51]
118	c.1520_1521del/p.(Val507Glufs*2)	Small deletion/ frameshift	*SLC20A2* exon 8	[52]
119	c.1523G>A/p.(Ser508Asn)	Missense	*SLC20A2* exon 8	[14]
120	c.1523+1G>A/p.(Gly312Valfs*8)	Splicing/ frameshift	*SLC20A2* intron 8	[27]
121	c.1524-2A>G/p.?	Splicing	*SLC20A2* intron 8	[14]
122	c.1527del/p.(Asn509Lysfs*7)	Small deletion/ frameshift	*SLC20A2* exon 9	[26]
123	c.1535G>A/p.(Gly512Asp)	Missense	*SLC20A2* exon 9	[53]
124	c.1567C>T/p.(Gln523*)	Nonsense	*SLC20A2* exon 9	[54]
125	c.1584del/p.(Glu529Lysfs*27)	Small deletion/ frameshift	*SLC20A2* exon 9	[55]
126	c.1618G>A/p.(Gly540Arg)	Missense	*SLC20A2* exon 9	[56]
127	c.1637_1638del/p.(Thr546Argfs*52)	Small deletion/ frameshift	*SLC20A2* exon 9	[14]
128	c.1640G>C/p.(Gly547Ala)	Missense	*SLC20A2* exon 9	[9]
129	c.1652G>A/p.(Trp551*)	Nonsense	*SLC20A2* exon 9	[27]
130	c.1656del/p.(Arg553Glufs*3)	Small deletion/ frameshift	*SLC20A2* exon 9	[57]
131	c.1663G>A/p.Val555Met	Missense	*SLC20A2* exon 9	[6]
**132**	**c.1669C>T/p.(Gln557*)**	**Nonsense**	***SLC20A2* exon 9**	**This study**
133	c.1673C>T/p.Thr558Ile	Missense	*SLC20A2* exon 9	[6, 9]
134	c.1685A > T/p.(Asp562Val)	Missense	*SLC20A2* exon 9	[58]
135	c.1703C>T/p.(Pro568Leu)	Missense	*SLC20A2* exon 9	[27]
136	c.1711G>A/p.Gly571Ser	Missense	*SLC20A2* exon 10	[6, 26]
137	c.1723G>A/p.Glu575Lys	Missense	*SLC20A2* exon 10	[6, 12]
138	c.1723G>T/p.(Glu575*)	Nonsense	*SLC20A2* exon 10	[59]
139	c.1753G>A/p.Ala585Thr	Missense	*SLC20A2* exon 10	[6, 31]
140	c.1755_1768del/p.(Asn587Serfs*7)	Small deletion/ frameshift	*SLC20A2* exon 10	[31]
141	c.1765G>A/p.Gly589Arg	Missense	*SLC20A2* exon 10	[6, 14]
142	c.1781C>T/p.(Thr594Ile)	Missense	*SLC20A2* exon 10	[60]
143	c.1784C>T/p.(Thr595Met)	Missense	*SLC20A2* exon 10	[12]
144	c.1786C>T/p.(His596Tyr)	Missense	*SLC20A2* exon 10	[61]
145	c.1787A>G/p.(His596Arg)	Missense	*SLC20A2* exon 10	[9]
146	c.1790G>A/p.Cys597Tyr	Missense	*SLC20A2* exon 10	[6, 62]

No.	Sequence change#	Variant type§	Involved regions	References
147	c.1794+1del/p.?	Splicing	*SLC20A2* intron 10	[63]
148	c.1794+1G>T/p.?	Splicing	*SLC20A2* intron 10	[9]
149	c.1794+1G>A/p.(Ser570Argfs*30)	Splicing/ frameshift	*SLC20A2* intron 10	[27]
150	c.1794+1G>C/p.?	Splicing	*SLC20A2* intron 10	[27]
151	c.1795-1698A>G/p.?	Splicing	*SLC20A2* intron 10	[23]
152	c.1795-1G>A/p.?	Splicing	*SLC20A2* intron 10	[22]
153	c.1801T>C/p.Ser601Pro	Missense	*SLC20A2* exon 11	[6]
154	c.1802C>G/p.(Ser601Trp)	Missense	*SLC20A2* exon 11	[12]
155	c.1802C>T/p.Ser601Leu	Missense	*SLC20A2* exon 11	[6, 12]
156	c.1810G>C/p.(Ala604Pro)	Missense	*SLC20A2* exon 11	[9]
157	c.1811C>T/p.(Ala604Val)	Missense	*SLC20A2* exon 11	[9]
158	c.1822_1848del/p.(Ile608_Trp616del)	Gross deletion	*SLC20A2* exon 11	[14]
159	c.1828_1831del/p.(Ser610Alafs*18)	Small deletion	*SLC20A2* exon 11	[27]
160	c.1848G>A/p.(Trp616*)	Nonsense	*SLC20A2* exon 11	[64]
161	c.1871T>A/p.(Val624Glu)	Missense	*SLC20A2* exon 11	[14]
162	c.1876T>A/p.Trp626Arg	Missense	*SLC20A2* exon 11	[6]
163	c.1876_1887dup/p.Trp626_Thr629dup	Small duplication	*SLC20A2* exon 11	[65]
164	c.1882G>A/p.Val628Met	Missense	*SLC20A2* exon 11	[6]
165	c.1909A>C/p.(Ser637Arg)	Missense	*SLC20A2* exon 11	[15]

PBC, primary brain calcification; *SLC20A2*, the solute carrier family 20 member 2 gene; UTR, untranslated region.

#The sequence change nomenclature refers to the reference sequence (GRCh37/hg19, NC_000008.10, NM_001257180.2, NP_001244109.1) following the Human Genome Variation Society Nomenclature (https://hgvs-nomenclature.org/stable/), in which the corresponding amino acid change is described after the slash "/".

§For the description of variant type:

(1) Frameshift includes small deletion or duplication/insertion with changes involving 20 bp or less leading to reading frame shift, as well as splicing variant;

(2) Gross deletion or duplication, referring to large deletion or duplication over 20 bp, may be described following the genomic or coding DNA reference sequence as reported in the reference articles.

REFERENCES

1. Pasanen P, Mäkinen J, Myllykangas L, Guerreiro R, Bras J, Valori M, et al. Primary familial brain calcification linked to deletion of 5' noncoding region of SLC20A2. Acta Neurol Scand. 2017;136(1):59-63. https://doi.org/10.1111/ane.12697 PMID: 27726124
2. David S, Ferreira J, Quenez O, Rovelet-Lecrux A, Richard A-C, Vérin M, et al. Identification of partial SLC20A2 deletions in primary brain calcification using whole-exome sequencing. Eur J Hum Genet. 2016;24(11):1630-4. https://doi.org/10.1038/ejhg.2016.50 PMID: 27245298
3. Sennfält S, Gustavsson P, Malmgren H, Gilland E, Almqvist H, Oscarson M, et al. Novel findings in a Swedish primary familial brain calcification cohort. J Neurol Sci. 2024;460:123020. https://doi.org/10.1016/j.jns.2024.123020 PMID: 38642488
4. Baker M, Strongosky AJ, Sanchez-Contreras MY, Yang S, Ferguson W, Calne DB, et al. SLC20A2 and THAP1 deletion in familial basal ganglia calcification with dystonia. Neurogenetics. 2014;15(1):23-30. https://doi.org/10.1007/s10048-013-0378-5 PMID: 24135862
5. Guo XX, Su HZ, Zou XH, Lai LL, Lu YQ, Wang C, et al. Identification of SLC20A2 deletions in patients with primary familial brain calcification. Clin Genet. 2019;96(1):53-60. https://doi.org/10.1111/cge.13540 PMID: 30891739
6. Chen S, Cen Z, Fu F, Chen Y, Chen X, Yang D, et al. Underestimated disease prevalence and severe phenotypes in patients with biallelic variants: a cohort study of primary familial brain calcification from China. Parkinsonism Relat Disord. 2019;64:211-9. https://doi.org/10.1016/j.parkreldis.2019.04.009 PMID: 31003906
7. Gagliardi M, Morelli M, Annesi G, Nicoletti G, Perrotta P, Pustorino G, et al. A new SLC20A2 mutation identified in southern Italy family with primary familial brain calcification. Gene. 2015;568(1):109-11. https://doi.org/10.1016/j.gene.2015.05.005 PMID: 25958344
8. Soh D, Lang A. Diffuse brain calcification, a novel SLC20A2 variant, vertical supranuclear gaze palsy, and systemic lupus erythematosus. Mov Disord Clin Pract. 2019;6(5):403-5. https://doi.org/10.1002/mdc3.12763 PMID: 31286012
9. Guo XX, Zou XH, Wang C, Yao XP, Su HZ, Lai LL, et al. Spectrum of SLC20A2, PDGFRB, PDGFB, and XPR1 mutations in a large cohort of patients with primary familial brain calcification. Hum Mutat. 2019;40(4):392-403. https://doi.org/10.1002/humu.23703 PMID: 30609140
10. Chen WJ, Yao XP, Zhang QJ, Ni W, He J, Li HF, et al. Novel SLC20A2 mutations identified in southern Chinese patients with idiopathic basal ganglia calcification. Gene. 2013;529(1):159-62. https://doi.org/10.1016/j.gene.2013.07.071 PMID: 23939468
11. Jensen MP, Spasic-Boskovic O, Rowe JB, Galton C, Allinson KSJ. Clinicopathological co-occurrence of Fahr's disease and dementia with Lewy bodies. Clin Neuropathol. 2020;39(5):227-31. https://doi.org/10.5414/np301267 PMID: 32145757

12. Wang C, Li Y, Shi L, Ren J, Patti M, Wang T, et al. Mutations in SLC20A2 link familial idiopathic basal ganglia calcification with phosphate homeostasis. Nat Genet. 2012;44(3):254-6. https://doi.org/10.1038/ng.1077 PMID: 22327515
13. Xiao C, Cassini T, Benavides D, Ebrahim A, Adams D, Toro C. Genomic diagnoses for ectopic intracerebral calcifications. Neurol Genet. 2023;9(5):e200083. https://doi.org/10.1212/nxg.0000000000200083 PMID: 37547187
14. Ramos EM, Carecchio M, Lemos R, Ferreira J, Legati A, Sears RL, et al. Primary brain calcification: an international study reporting novel variants and associated phenotypes. Eur J Hum Genet. 2018;26(10):1462-77. https://doi.org/10.1038/s41431-018-0185-4 PMID: 29955172
15. Yamada M, Tanaka M, Takagi M, Kobayashi S, Taguchi Y, Takashima S, et al. Evaluation of SLC20A2 mutations that cause idiopathic basal ganglia calcification in Japan. Neurology. 2014;82(8):705-12. https://doi.org/10.1212/wnl.0000000000000143 PMID: 24463626
16. Rubino E, Giorgio E, Godani M, Grosso E, Zibetti M, Lopiano L, et al. Three novel missense mutations in SLC20A2 associated with idiopathic basal ganglia calcification. J Neurol Sci. 2017;377:62-4. https://doi.org/10.1016/j.jns.2017.03.053 PMID: 28477710
17. Arteche-López A, Álvarez-Mora MI, Sánchez Calvin MT, Lezana Rosales JM, Palma Milla C, Gómez Rodríguez MJ, et al. Biallelic variants in genes previously associated with dominant inheritance: CACNA1A, RET and SLC20A2. Eur J Hum Genet. 2021;29(10):1520-6. https://doi.org/10.1038/s41431-021-00919-5 PMID: 34267336
18. Røsby O, Legati A, Coppola G. Primary familial brain calcification in a Norwegian family, caused by a novel SLC20A2 gene mutation. J Neurol. 2016;263(3):594-6. https://doi.org/10.1007/s00415-016-8033-3 PMID: 26860091
19. Ding Y, Dong HQ. A novel SLC20A2 mutation associated with familial idiopathic basal ganglia calcification and analysis of the genotype-phenotype association in Chinese patients. Chin Med J (Engl). 2018;131(7):799-803. https://doi.org/10.4103/0366-6999.228245 PMID: 29578123
20. Paucar M, Almqvist H, Björkhem I, Svenningsson P. Hyperkinesias and echolalia in primary familial brain calcification. Ann Neurol. 2021;89(2):418-9. https://doi.org/10.1002/ana.25955 PMID: 33170525
21. Koyama S, Sato H, Kobayashi R, Kawakatsu S, Kurimura M, Wada M, et al. Clinical and radiological diversity in genetically confirmed primary familial brain calcification. Sci Rep. 2017;7(1):12046. https://doi.org/10.1038/s41598-017-11595-1 PMID: 28935882
22. Giorgio E, Garelli E, Carando A, Bellora S, Rubino E, Quarello P, et al. Design of a multiplex ligation-dependent probe amplification assay for SLC20A2: identification of two novel deletions in primary familial brain calcification. J Hum Genet. 2019;64(11):1083-90. https://doi.org/10.1038/s10038-019-0668-3 PMID: 31501477
23. Zhao M, Cheng X, Chen L, Zeng YH, Lin KJ, Li YL, et al. Antisense oligonucleotides enhance SLC20A2 expression and suppress brain calcification in a humanized mouse model. Neuron.

2024;112(19):3278-94.e7. https://doi.org/10.1016/j.neuron.2024.07.013 PMID: 39121859

24. Kasuga K, Konno T, Saito K, Ishihara A, Nishizawa M, Ikeuchi T. A Japanese family with idiopathic basal ganglia calcification with novel SLC20A2 mutation presenting with late-onset hallucination and delusion. J Neurol. 2014;261(1):242-4. https://doi.org/10.1007/s00415-013-7205-7 PMID: 24323245
25. Hozumi I, Kurita H, Ozawa K, Furuta N, Inden M, Sekine SI, et al. Inorganic phosphorus (Pi) in CSF is a biomarker for SLC20A2-associated idiopathic basal ganglia calcification (IBGC1). J Neurol Sci. 2018;388:150-4. https://doi.org/10.1016/j.jns.2018.03.014 PMID: 29627011
26. Nicolas G, Pottier C, Charbonnier C, Guyant-Maréchal L, Le Ber I, Pariente J, et al. Phenotypic spectrum of probable and genetically-confirmed idiopathic basal ganglia calcification. Brain. 2013;136(Pt 11):3395-407. https://doi.org/10.1093/brain/awt255 PMID: 24065723
27. Hsu SC, Sears RL, Lemos RR, Quintáns B, Huang A, Spiteri E, et al. Mutations in SLC20A2 are a major cause of familial idiopathic basal ganglia calcification. Neurogenetics. 2013;14(1):11-22. https://doi.org/10.1007/s10048-012-0349-2 PMID: 23334463
28. Zhang Y, Guo X, Wu A. Association between a novel mutation in SLC20A2 and familial idiopathic basal ganglia calcification. PLoS One. 2013;8(2):e57060. https://doi.org/10.1371/journal.pone.0057060 PMID: 23437308
29. Magistrelli L, Croce R, De Marchi F, Basagni C, Carecchio M, Nasuelli N, et al. Expanding the genetic spectrum of primary familial brain calcification due to SLC2OA2 mutations: a case series. Neurogenetics. 2021;22(1):65-70. https://doi.org/10.1007/s10048-021-00634-9 PMID: 33471268
30. Nan H, Takaki R, Ichinose Y, Tsuchiya M, Koh K, Hanyu S, et al. Novel SLC20A2 mutation in primary familial brain calcification with disturbance of sustained phonation and orofacial apraxia. J Neurol Sci. 2018;390:1-3. https://doi.org/10.1016/j.jns.2018.03.031 PMID: 29801865
31. Lemos RR, Ramos EM, Legati A, Nicolas G, Jenkinson EM, Livingston JH, et al. Update and mutational analysis of SLC20A2: a major cause of primary familial brain calcification. Hum Mutat. 2015;36(5):489-95. https://doi.org/10.1002/humu.22778 PMID: 25726928
32. Zhang Q, Li Z, Sun H, Zhang S, Zhang J, Wang Y, et al. Generation of induced pluripotent stem cell line (ZZUi0012-A) from a patient with Fahr’s disease caused by a novel mutation in SLC20A2 gene. Stem Cell Res. 2019;35:101395. https://doi.org/10.1016/j.scr.2019.101395 PMID: 30776674
33. Sun D, Wang Y, Wang J, Wang S, Zhu L, Xia K, et al. Primary familial brain calcification presenting with parkinsonism and motor complications caused by a novel SLC20A2 variant: a case report. Front Neurol. 2024;15:1382534. https://doi.org/10.3389/fneur.2024.1382534 PMID: 39036637
34. Grütz K, Volpato CB, Domingo A, Alvarez-Fischer D, Gebert U, Schifferle G, et al. Primary familial brain calcification in the ‘IBGC2’ kindred: all linkage roads lead to SLC20A2. Mov Disord. 2016;31(12):1901-4. https://doi.org/10.1002/mds.26768 PMID: 27671522

35. Reyes NGD, Lang AE. Vertical supranuclear gaze palsy in primary familial brain calcification associated with a novel SLC20A2 mutation. Mov Disord Clin Pract. 2022;10(3):501-3. https://doi.org/10.1002/mdc3.13604 PMID: 36989014
36. Nishii K, Shimogawa R, Kurita H, Inden M, Kobayashi M, Toyoshima I, et al. Partial reduced Pi transport function of PiT-2 might not be sufficient to induce brain calcification of idiopathic basal ganglia calcification. Sci Rep. 2019;9(1):17288. https://doi.org/10.1038/s41598-019-53401-0 PMID: 31754123
37. Shen Y, Shu S, Ren Y, Xia W, Chen J, Dong L, et al. Case report: two novel frameshift mutations in SLC20A2 and one novel splice donor mutation in PDGFB associated with primary familial brain calcification. Front Genet. 2021;12:643452. https://doi.org/10.3389/fgene.2021.643452 PMID: 34025715
38. Bezerra DP, Keasey M, Oliveira JRM. MiR-9-5p down-regulates PiT2, but not PiT1 in human embryonic kidney 293 cells. J Mol Neurosci. 2017;62(1):28-33. https://doi.org/10.1007/s12031-017-0906-0 PMID: 28303467
39. Sakai K, Ishida C, Hayashi K, Tsuji N, Kannon T, Hosomichi K, et al. Familial idiopathic basal ganglia calcification with a heterozygous missense variant (c.902C>T/p.P307L) in SLC20A2 showing widespread cerebrovascular lesions. Neuropathology. 2022;42(2):126-33. https://doi.org/10.1111/neup.12781 PMID: 35026865
40. Ichikawa Y, Tanaka M, Kurita E, Nakajima M, Tanaka M, Oishi C, et al. Novel SLC20A2 variant in a Japanese patient with idiopathic basal ganglia calcification-1 (IBGC1) associated with dopa-responsive parkinsonism. Hum Genome Var. 2019;6:44. https://doi.org/10.1038/s41439-019-0073-7 PMID: 31645982
41. Knowles JK, Santoro JD, Porter BE, Baumer FM. Refractory focal epilepsy in a paediatric patient with primary familial brain calcification. Seizure. 2018;56:50-2. https://doi.org/10.1016/j.seizure.2018.02.001 PMID: 29448117
42. Cassinari K, Rovelet-Lecrux A, Tury S, Quenez O, Richard AC, Charbonnier C, et al. Haploinsufficiency of the primary familial brain calcification gene SLC20A2 mediated by disruption of a regulatory element. Mov Disord. 2020;35(8):1336-45. https://doi.org/10.1002/mds.28090 PMID: 32506582
43. Zhu M, Fang C, Li X, Zhou M, Wan H, Hong D. Clinical features of familial idiopathic basal ganglia calcification caused by a novel mutation in the SLC20A2 gene. Zhonghua Yi Xue Yi Chuan Xue Za Zhi. 2015;32(1):64-8. https://doi.org/10.3760/cma.j.issn.1003-9406.2015.01.014 PMID: 25636102
44. Li M, Fu Q, Xiang L, Zheng Y, Ping W, Cao Y. SLC20A2-associated idiopathic basal ganglia calcification (Fahr disease): a case family report. BMC Neurol. 2022;22(1):438. https://doi.org/10.1186/s12883-022-02973-y PMID: 36397039
45. Taglia I, Mignarri A, Olgiati S, Menci E, Petrocelli PL, Breedveld GJ, et al. Primary familial brain

calcification: genetic analysis and clinical spectrum. Mov Disord. 2014;29(13):1691-5. https://doi.org/10.1002/mds.26053 PMID: 25284758

46. Boudriot ET, Hainc N, Balint B. Basal ganglia and prominent cortical contouring calcification in SLC20A2-related primary familial brain calcification. Mov Disord Clin Pract. 2025;12(2):258-9. https://doi.org/10.1002/mdc3.14299 PMID: 39644228
47. Ferreira JB, Pimentel L, Keasey MP, Lemos RR, Santos LM, Oliveira MF, et al. First report of a de novo mutation at SLC20A2 in a patient with brain calcification. J Mol Neurosci. 2014;54(4):748-51. https://doi.org/10.1007/s12031-014-0357-9 PMID: 24969325
48. Kuroi Y, Akagawa H, Yoneyama T, Kikuchi A, Maegawa T, Onda H, et al. Novel SLC20A2 mutation in a Japanese pedigree with primary familial brain calcification. J Neurol Sci. 2019;399:183-5. https://doi.org/10.1016/j.jns.2019.02.033 PMID: 30826713
49. Oliva M, Capaldo G, D'Amico A, Colavito D, Elefante A, Straccia G, et al. A novel SLC20A2 gene mutation causing primary familial brain calcification in an Ukrainian patient. Neurol Sci. 2019;40(6):1283-5. https://doi.org/10.1007/s10072-018-3684-8 PMID: 30607525
50. Lemos RR, Oliveira MF, Oliveira JRM. Reporting a new mutation at the SLC20A2 gene in familial idiopathic basal ganglia calcification. Eur J Neurol. 2013;20(3):e43-4. https://doi.org/10.1111/ene.12044 PMID: 23406454
51. Kumar N, Jog M. Fahr's disease presenting as late-onset levodopa-responsive parkinsonism. Can J Neurol Sci. 2017;44(3):322-3. https://doi.org/10.1017/cjn.2016.428 PMID: 28091340
52. Rubino E, Giorgio E, Gallone S, Pinessi L, Orsi L, Gentile S, et al. Novel mutation of SLC20A2 in an Italian patient presenting with migraine. J Neurol. 2014;261(10):2019-21. https://doi.org/10.1007/s00415-014-7475-8 PMID: 25178512
53. Salamon A, Zádori D, Ujfalusi A, Szpisjak L, Lukács M, Bihari B, et al. Hereditary and non-hereditary etiologies associated with extensive brain calcification: case series. Metab Brain Dis. 2021;36(7):2131-9. https://doi.org/10.1007/s11011-021-00790-9 PMID: 34287746
54. Zhan FX, Tian WT, Zhang C, Zhu ZY, Wang SG, Huang XJ, et al. Primary familial brain calcification presenting as paroxysmal kinesigenic dyskinesia: genetic and functional analyses. Neurosci Lett. 2020;714:134543. https://doi.org/10.1016/j.neulet.2019.134543 PMID: 31618668
55. Coppola A, Hernandez-Hernandez L, Balestrini S, Krithika S, Moran N, Hale B, et al. Cortical myoclonus and epilepsy in a family with a new SLC20A2 mutation. J Neurol. 2020;267(8):2221-7. https://doi.org/10.1007/s00415-020-09821-4 PMID: 32274582
56. Brighina L, Saracchi E, Ferri F, Gagliardi M, Tarantino P, Morzenti S, et al. Fahr's disease linked to a novel SLC20A2 gene mutation manifesting with dynamic aphasia. Neurodegener Dis. 2014;14(3):133-8. https://doi.org/10.1159/000365216 PMID: 25348593
57. Reyes NGD, Lang AE. Adult-onset tourettism in SLC20A2-associated primary familial brain calcification. Mov Disord Clin Pract. 2023;10(7):1152-4. https://doi.org/10.1002/mdc3.13766 PMID: 37476312

58. Zhu SH, Peng JJ, Li KF, Peng JC, Li J. Non-ketotic hyperglycaemic hemichorea-hemiballismus with Fahr's disease in a Chinese family: a case report. Int J Neurosci. 2023;133(11):1242-6. https://doi.org/10.1080/00207454.2022.2074846 PMID: 35510430
59. Bu W, Hou L, Zhu M, Zhang R, Zhang X, Zhang X, et al. SLC20A2-related primary familial brain calcification with purely acute psychiatric symptoms: a case report. BMC Neurol. 2022;22(1):265. https://doi.org/10.1186/s12883-022-02798-9 PMID: 35850697
60. Sharma R, Stitt D. Novel likely pathogenic SLC20A variant in primary familial brain calcification. BMJ Case Rep. 2022;15(3):e245909. https://doi.org/10.1136/bcr-2021-245909 PMID: 35236675
61. Wang L, Ma J, Che X. A novel SLC20A2 mutation presenting with paroxysmal kinesigenic dyskinesia and epilepsy in a Chinese patient: a case report. Acta Neurol Belg. 2023;123(6):2379-82. https://doi.org/10.1007/s13760-023-02182-5 PMID: 36701080
62. Liu X, Ma G, Zhao Z, Mao F, Tang J, Li X, et al. Novel mutation of SLC20A2 in a Chinese family with primary familial brain calcification. J Neurol Sci. 2016;360:1-3. https://doi.org/10.1016/j.jns.2015.11.036 PMID: 26723961
63. McKenna MC, Redmond J, Bradley D, Bede P. Teaching neuroimage: primary familial brain calcification in SLC20A2 genotype. Neurology. 2022;99(22):1008-9. https://doi.org/10.1212/wnl.0000000000201343 PMID: 36127139
64. Takeuchi T, Muraoka K, Yamada M, Nishio Y, Hozumi I. Living with idiopathic basal ganglia calcification 3: a qualitative study describing the lives and illness of people diagnosed with a rare neurological disease. SpringerPlus. 2016;5(1):1713. https://doi.org/10.1186/s40064-016-3390-z PMID: 27777849
65. Taglia I, Formichi P, Battisti C, Peppoloni G, Barghigiani M, Tessa A, et al. Primary familial brain calcification with a novel SLC20A2 mutation: analysis of PiT-2 expression and localization. J Cell Physiol. 2018;233(3):2324-31. https://doi.org/10.1002/jcp.26104 PMID: 28722801
