# Supplementary material for: A novel SLC20A2 nonsense variant and mechanistic studies of primary brain calcification
Source: PLoS One. 2026 Apr 17;21(4):e0346635. doi: 10.1371/journal.pone.0346635 (PMC13089887; doi:10.1371/journal.pone.0346635)

Subject: The raw Western blotting images of wild-type and variant-type PiT2 in SH-SY5Y cells
Loading order: from left to right (Marker, Control, WT, S113*, Q557*, A585T, S601W, and Marker)
Method of images captured: Chemiluminescence with a ChemiDoc MP Imaging System (Bio-Rad, USA)
Figure panel generated from the original images: Fig 2B (the upper part)

PiT2

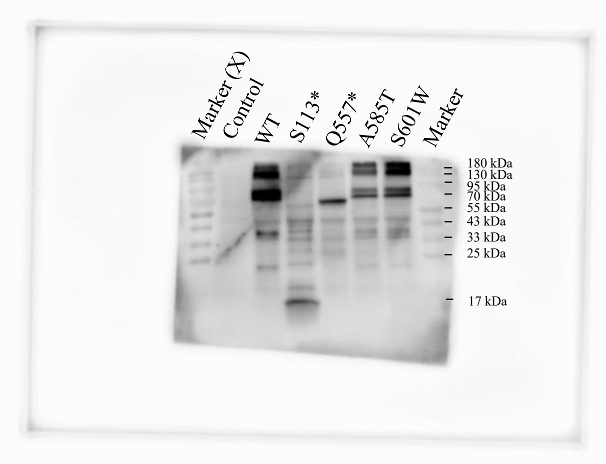

β-actin

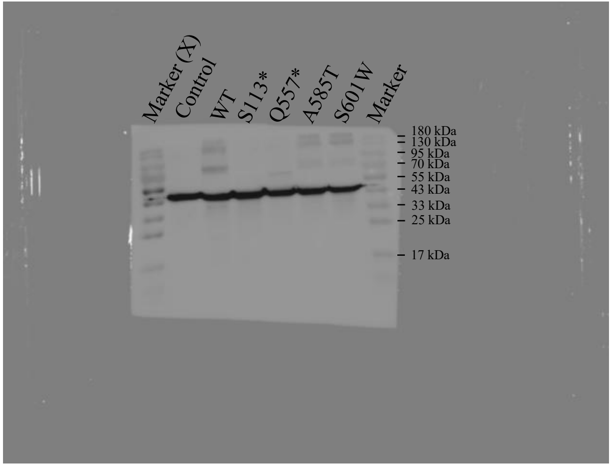

Biosharp BL712A
Protein Marker
(10-180 kDa)

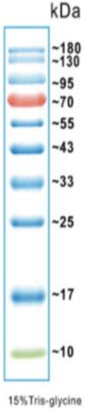

Subject: The raw Western blotting images of wild-type and variant-type PiT2 in HEK293T cells
Loading order: from left to right (Control, WT, S113*, Q557*, A585T, S601W, and Marker)
Method of images captured: Chemiluminescence with a ChemiDoc MP Imaging System (Bio-Rad, USA)
Figure panel generated from the original images: Fig 2B (the lower part)

Biosharp BL712A
Protein Marker
(10-180 kDa)

PiT2

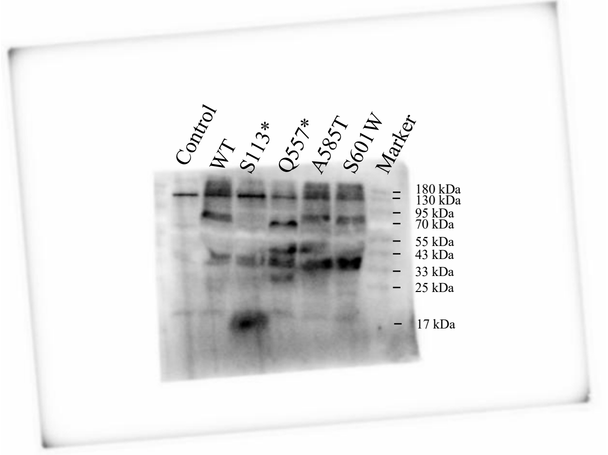

β-actin

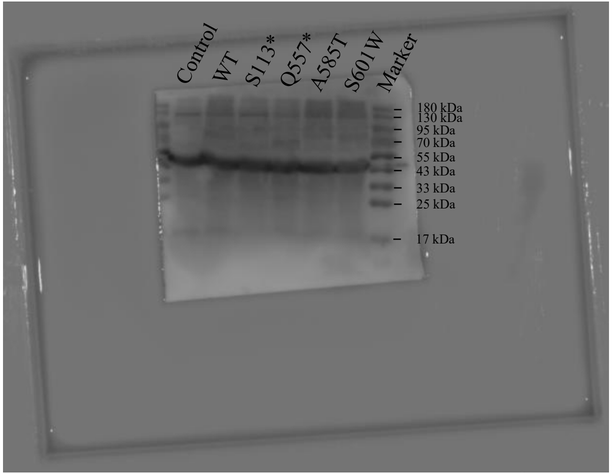

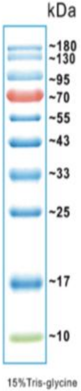

Supplement: S1 Raw Images — (PDF) [file pone.0346635.s002.pdf]
